# Prognostic Value of BUB1 for Predicting Non-Muscle-Invasive Bladder Cancer Progression

**DOI:** 10.3390/ijms222312756

**Published:** 2021-11-25

**Authors:** Xuan-Mei Piao, Chaelin You, Young Joon Byun, Ho Won Kang, Junho Noh, Jaehyun Lee, Hee Youn Lee, Kyeong Kim, Won Tae Kim, Seok Joong Yun, Sang-Cheol Lee, Kyuho Kang, Yong-June Kim

**Affiliations:** 1 Department of Urology, College of Medicine, Chungbuk National University, Cheongju 28644, Korea; phm1013@hotmail.com (X.-M.P.); lenic0819@naver.com (Y.J.B.); howon98@naver.com (H.W.K.); wtkimuro@chungbuk.ac.kr (W.T.K.); sjyun@chungbuk.ac.kr (S.J.Y.); lscuro@chungbuk.ac.kr (S.-C.L.); 2 Department of Biological Sciences and Biotechnology, Chungbuk National University, Cheongju 28644, Korea; clyu104@cbnu.ac.kr (C.Y.); shwnsgh9@cbnu.ac.kr (J.N.); jellyjaelee@cbnu.ac.kr (J.L.); 3 Department of Urology, Chungbuk National University Hospital, Cheongju 28644, Korea; leeheeyoun1@gmail.com (H.Y.L.); uro_kk@naver.com (K.K.)

**Keywords:** non-muscle-invasive bladder cancer, prognosis, *BUB1*, G2/M transition, cell cycle

## Abstract

Non-muscle-invasive bladder cancer (NMIBC) is a common disease with a high recurrence rate requiring lifetime surveillance. Although NMIBC is not life-threatening, it can progress to muscle-invasive bladder cancer (MIBC), a lethal form of the disease. The management of the two diseases differs, and patients with MIBC require aggressive treatments such as chemotherapy and radical cystectomy. NMIBC patients at a high risk of progression benefit from early immediate cystectomy. Thus, identifying concordant markers for accurate risk stratification is critical to predict the prognosis of NMIBC. Candidate genetic biomarkers associated with NMIBC prognosis were screened by RNA-sequencing of 24 tissue samples, including 16 NMIBC and eight normal controls, and by microarray analysis (GSE13507). Lastly, we selected and investigated a mitotic checkpoint serine/threonine kinase, BUB1, that regulates chromosome segregation during the cell cycle. *BUB1* gene expression was tested in 86 NMIBC samples and 15 controls by real-time qPCR. The performance of *BUB1* as a prognostic biomarker for NMIBC was validated in the internal Chungbuk cohort (GSE13507) and the external UROMOL cohort (E-MTAB-4321). *BUB1* expression was higher in NMIBC patients than in normal controls (*p* < 0.05), and the overexpression of *BUB1* was correlated with NMIBC progression (log-rank test, *p* = 0.007). In in vitro analyses, *BUB1* promoted the proliferation of bladder cancer cells by accelerating the G2/M transition of the cell cycle. Conclusively, *BUB1* modulates the G2/M transition to promote the proliferation of bladder cancer cells, suggesting that it could serve as a prognostic marker in NMIBC.

## 1. Introduction

Aneuploidy is the presence of an aberrant number of chromosomes in a cell. It is the result of chromosomal instability, a hallmark of cancer [1]. Defects in the mitotic spindle checkpoint contribute to chromosome instability and aneuploidy in several cancers [2,3,4]. Budding uninhibited by benzimidazole 1 (BUB1) is a well-characterized component of the spindle checkpoint that has versatile and distinct functions during the cell cycle [5]. BUB1 plays a role in oncogenesis, as indicated by the occurrence of *BUB1* mutations, as well as differential *BUB1* gene and protein expression in cancer tissues and cell lines [5]. *BUB1* is downregulated in sarcomas, lymphomas, and lung tumors, whereas *BUB1* upregulation is associated with liver cancer [6]. *BUB1* expression is correlated with unfavorable prognosis in patients with breast cancer and liver cancer [7,8,9]. In bladder cancer (BCa), weighted gene co-expression network analysis showed that *BUB1* is upregulated in high-grade BCa [10]; however, the roles of *BUB1* in BCa remain unclear.

BCa has a high morbidity rate, with 1,806,590 cases diagnosed in 2020 in the United States [11]. More than 70% of newly diagnosed BCa cases are non-muscle-invasive bladder cancer (NMIBC). Although these tumors are not aggressive, their high recurrence rates require lifetime monitoring, which is costly [12]. NMIBC can progress to muscle-invasive bladder cancer (MIBC), which is a fatal disease with a 5-year overall survival rate of <50% despite active intervention [13,14]. Thus, predicting the prognosis of NMIBC patients is critical to designing optimal treatment strategies. Clinical staging and grading systems, along with histopathological parameters, such as tumor size and tumor multiplicity, remain the “gold standard” for prognostic prediction of BCa [15]. However, these parameters are not sufficient to describe tumor behavior in clinical practice. The clinical use of prospective prognostic biomarkers may improve our understanding of the pathobiology of the disease to design effective surveillance methods and refine treatment strategies [16].

Advances in high-throughput techniques, such as microarray and RNA-sequencing (RNA-seq), together with well-established databases, such as the Cancer Genome Atlas (TCGA) and the public genomic data repository Gene Expression Omnibus (GEO), have uncovered new transcriptional landscapes, encouraging researchers to identify molecular markers for cancer diagnosis and prognosis [17]. Here, we performed RNA-seq analysis and identified *BUB1* as a differentially expressed gene in NMIBC and normal adjacent tissue (NAT). We showed that the overexpression of *BUB1* was correlated with poor clinical outcomes in patients of NMIBC. Real-time quantitative reverse transcription PCR (RT-qPCR) confirmed that *BUB1* upregulation in NMIBC patients was associated with higher progression rates. The value of *BUB1* as a prospective prognostic marker of NMIBC was validated in two published cohorts, which confirmed that NMIBC patients with high *BUB1* gene expression had higher progression rates. In addition, we found that *BUB1* promotes BCa cell proliferation by accelerating cell cycle G2/M transition. Taken together, the results indicate that *BUB1* could be a prospective marker of NMIBC progression and a potential novel therapeutic target.

## 2. Results

### 2.1. Screening Test

#### 2.1.1. Transcriptome Profiles in Patients with NMIBC

RNA-seq analysis of 24 bladder tissue samples, including 16 NMIBC and eight normal controls, was performed to identify the gene expression pattern in NMIBC. The heatmap depicted 1178 differentially expressed genes (DEGs) with 270 upregulated and 908 downregulated genes in NMIBC compared with NAT (FDR < 0.01, >2-fold differences in expression; Supplementary Figure S1A). Gene ontology (GO) analysis in biological processes revealed the functional profiles of DEGs, indicating that genes with higher transcription levels in NMIBC than in NAT were enriched in the following GO terms: cell division, DNA conformation change, regulation of cell cycle progression, and DNA repair. Downregulated genes were enriched in the following GO terms: extracellular matrix organization, cell–substrate adhesion, positive regulation of locomotion, and muscle structure development (Supplementary Figure S1B). These processes are well-known alterations in BCa related to tumor development and progression. Gene set enrichment analysis (GSEA) showed that upregulated genes in NMIBC patients were associated with cell cycle-related processes (Supplementary Figure S1C); the core genes enriched in the cell cycle pathway are shown in Supplementary Figure S1D. Overall, the results indicate that genes involved in the regulation of the cell cycle play crucial roles in NMIBC. 

#### 2.1.2. Comparison of DEGs in Two Different Cohorts

The intersection of 270 upregulated genes in NMIBC patients versus NATs from RNA-seq analysis, 481 upregulated genes in NMIBC versus normal controls from the GSE13507 dataset, and 527 upregulated genes in MIBC versus NMIBC from GSE13507 was determined to obtain target outlier genes for NMIBC prognosis. Accordingly, 19 upregulated genes overlapped among the three categories, suggesting that these genes are the most promising candidates for BCa. GO analysis revealed that the 19 genes were cell cycle-related genes (Supplementary Figure S2). Among them, *BUB1*, a spindle checkpoint regulator, was selected for further evaluation of its role in NMIBC pathogenesis and prognosis. The expression of *BUB1* in patients with NMIBC and NATs from RNA-seq analysis is shown in Supplementary Figure S3.

### 2.2. Training Test

#### 2.2.1. *BUB1* mRNA Expression in NMIBC Tissues

**Figure 1:**
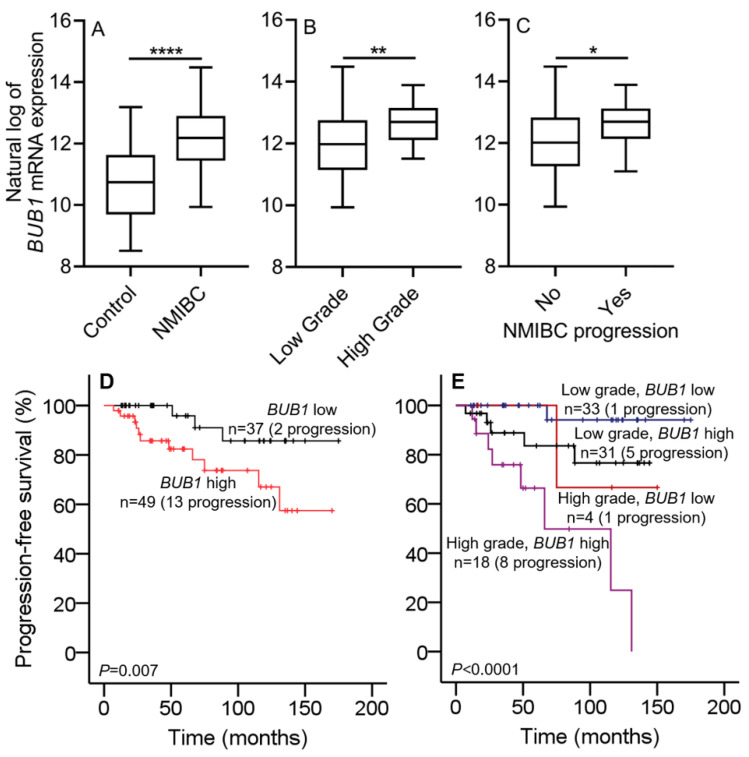
Gene expression of BUB1 in the training cohort. (**A**) *BUB1* mRNA expression was significantly higher in NMIBC patients than in normal controls. (**B**) *BUB1* mRNA expression was significantly higher in high-grade NMIBC patients than in low-grade NMIBC patients. (**C**) *BUB1* mRNA expression was lower in NMIBC patients who did not progress to MIBC than in patients with progression. (**D**) Progression-free survival plot of NMIBC patients. NMIBC patients were divided into two groups according to the expression of *BUB1* (upper 50th percentile and lower 50th percentile groups). The progression-free survival of NMIBC patients was significantly higher in the lower *BUB1* expression group (log-rank test, *p* = 0.007). (**E**) Progression-free survival of NMIBC patients according to combination indexes (tumor grade + *BUB1* expression). NMIBC patients were divided into four groups (highest quartile, middle two quartiles, and lowest quartile groups) according to tumor grade and *BUB1* expression levels. High-grade NMIBC patients with higher *BUB1* expression had a shorter progression-free survival than the middle two quartiles and the lowest quartile groups (log-rank test, *p* < 0.0001). NMIBC, non-muscle-invasive bladder cancer; MIBC, muscle-invasive bladder cancer. Control samples consisted of normal bladder mucosa samples. *p*-value determined by the Mann–Whitney test. * *p* < 0.05, ** *p* < 0.01, **** *p* < 0.0001.

The mRNA expression of *BUB1* in NMIBC tissues was detected by real-time RT-qPCR, which showed that *BUB1* expression was higher in NMIBC tissues than in controls (normal bladder mucosae; *p* < 0.0001; Figure 1A). *BUB1* expression varied between NMIBC patients with different grades and prognoses. *BUB1* expression was significantly higher in high-grade NMIBC patients than in those with low-grade tumors (*p* < 0.01; Figure 1B). Moreover, *BUB1* was considerably overexpressed in NMIBC patients with progression when compared to those patients without progression (*p* < 0.05; Figure 1C). These results suggest that *BUB1* is a prognostic indicator of NMIBC.

#### 2.2.2. *BUB1* mRNA Expression Correlates with NMIBC Prognosis

Survival analyses were performed to evaluate the prognostic power of *BUB1* in NMIBC. NMIBC patients were classified into two groups based on median cut-off values of *BUB1* (*BUB1* high and *BUB1* low). Univariate and multivariate Cox regression analyses of the real-time RT-qPCR cohort indicated that *BUB1* expression may be an independent predictor of progression in NMIBC patients [hazard ratio (HR), 4.642; 95% confidence interval (CI), 1.021–21.097; *p* = 0.047], and the HR was similar to that for tumor grade (HR, 4.629; 95% CI, 1.593–13.450; *p* = 0.005; Table 1). The Kaplan–Meier survival plot showed NMIBC patients with decreased *BUB1* expression had a lower risk of progression than those with increased *BUB1* expression (log-rank test, *p* = 0.007; Figure 1D). Kaplan–Meier analysis combining the 2004 WHO grading system with *BUB1* expression demonstrated that low-grade NMIBC patients with low *BUB1* expression had the lowest rate of progression, whereas high-grade NMIBC patients with high *BUB1* expression had the highest rate of progression (log-rank test, *p* < 0.0001; Figure 1E).

### 2.3. Validation Test

The expression levels of *BUB1* in NMIBC were validated using public data (GSE13507 and E-MTAT-4321).

#### 2.3.1. *BUB1* mRNA Expression in the GSE13507 Dataset: Internal Validation

**Figure 2:**
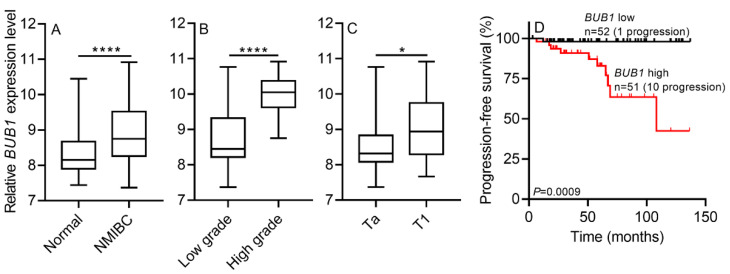
BUB1 gene expression in the internal validation cohort (GSE13507). *BUB1* expression was higher in patients with NMIBC than in normal controls (**A**), in high-grade NMIBC patients than in low-grade NMIBC patients (**B**), and in stage T1 than in stage Ta patients (**C**) from the CBNUH microarray (GSE13507 dataset) cohort. (**D**) A Kaplan–Meier survival plot demonstrated that NMIBC patients with lower *BUB1* expression had a lower rate of progression (log-rank test, *p* = 0.0009). CBNUH, Chungbuk National University Hospital; NAT, normal adjacent tumor; NMIBC, non-muscle-invasive bladder cancer. Results are expressed as the mean with 95% CI. *p*-values were determined by Welch’s *t*-test. **** *p* < 0.0001 and * *p* < 0.05.

*BUB1* mRNA expression was significantly higher in NMIBC than in normal controls in the GSE13507 cohort (Figure 2A), indicating that the upregulation of *BUB1* may be an important event during NMIBC pathogenesis. *BUB1* expression was significantly higher in NMIBC patients with T1 stage and a high grade than in those with Ta stage and a low grade (both *p* < 0.05; Figure 2B,C). Because tumor stage and grade are the most common predictors of tumor prognosis, these results suggest that the upregulation of *BUB1* is a prognostic indicator of NMIBC. Moreover, univariate Cox regression analysis demonstrated that the *BUB1* gene was an independent prognostic variable in NMIBC progression-free survival (PFS) (HR, 15; 95% CI, 1.9–115; *p* = 0.011; Table 2). The survival plot depicted that higher *BUB1* expression in NMIBC patients correlated with worse PFS (log-rank test, *p* = 0.0009; Figure 2D).

#### 2.3.2. *BUB1* mRNA Expression in the E-MTAT-4321 Dataset: External Validation

**Figure 3:**
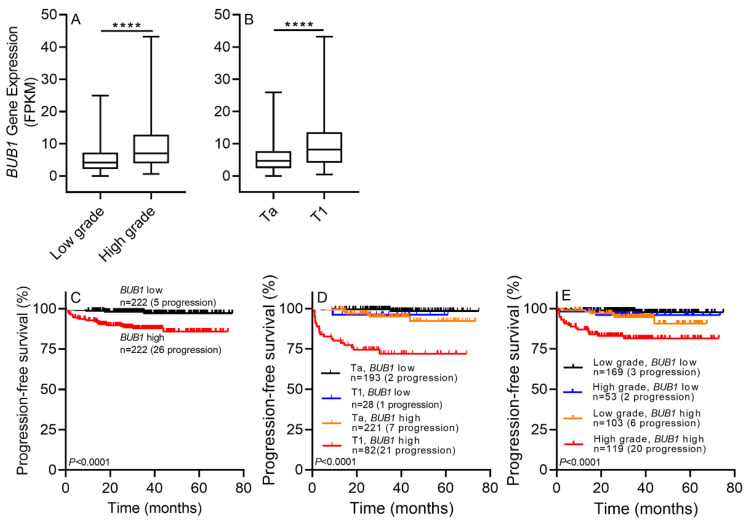
*BUB1* gene expression in the external validation cohort (E-MTAB-4321). *BUB1* expression was higher in high-grade NMIBC patients than in low-grade NMIBC patients (**A**) and in stage T1 than in stage Ta patients (**B**) from the UROMOL (E-MTAB-4321 dataset) cohort. (**C**) The Kaplan–Meier survival plot demonstrated that NMIBC patients with lower *BUB1* expression had a lower rate of progression (log-rank test, *p* < 0.0001). (**D**) The Kaplan–Meier survival plot demonstrated that NMIBC patients with Ta stage and lower *BUB1* expression had the lowest progression rates (log-rank test, *p* < 0.0001). (**E**) The Kaplan–Meier survival plot demonstrated that NMIBC patients with a low grade and lower *BUB1* expression had the lowest progression rates (log-rank test, *p* < 0.0001). FPKM, fragments per kilobase of transcripts per million mapped reads; NAT, normal adjacent tissue; NMIBC, non-muscle-invasive bladder cancer. Results are expressed as the mean with 95% CI. *p*-values were determined by Welch’s *t*-test. **** *p* < 0.0001.

Similar results were obtained in the E-MTAT-4321 cohort, which showed that high-grade or T1-stage NMIBC patients had higher *BUB1* expression levels than low-grade or Ta-stage patients (both *p* < 0.0001; Figure 3A,B). However, there were no controls in this cohort, and a comparison between NMIBC and controls could not be performed. Univariate Cox regression analysis identified the prognostic significance of *BUB1* in NMIBC, with an HR for PFS of 5.7 (95% CI, 2.2–15; *p* < 0.001; Table 2). PFS analysis showed that the progression rate was declined when *BUB1* expression was reduced (log-rank test, both *p* < 0.0001; Figure 3C). After incorporating tumor stage and grade with *BUB1* expression in the E-MTAT-4321 cohort, NMIBC patients in the *BUB1* high-grade and T1 stage group showed the worst PFS, whereas those in the *BUB1* low-grade and Ta stage group had the best PFS (log-rank test, *p* < 0.0001; Figure 3D). Patients in the *BUB1* high-expression, high-grade group had the worst PFS, whereas those in the *BUB1* low-expression, low-grade group had the best PFS (log-rank test, *p* < 0.0001; Figure 3E).

### 2.4. BUB1 Inhibits G2/M Cell Cycle Arrest in Human BCa Cells

**Figure 4:**
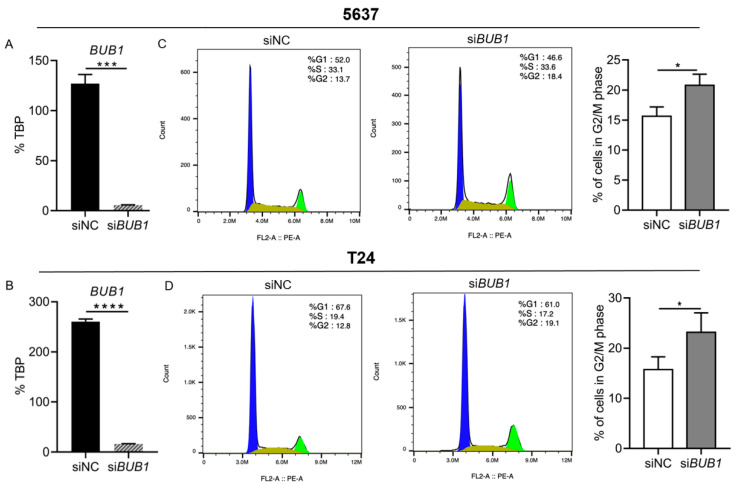
*BUB1* promotes bladder cancer cell proliferation by accelerating the G2/M transition of the cell cycle. *BUB1* expression was inhibited by siRNA transfection in 5637 (**A**) and T24 cells (**B**). Cell cycle progression assay of 5637 (**C**) and T24 (**D**) cells transfected with NC or *BUB1* siRNA. Cell cycle analysis was performed by flow cytometry. Error bar: mean value with SEM. NC, non-targeting control; SEM, standard error of the mean. *p*-values were determined by Welch’s *t*-test. * *p* < 0.05, *** *p* < 0.001, **** *p* < 0.0001.

Two BCa cell lines, 5637 and T24, were used to evaluate the effects of *BUB1* on cell cycle progression. The expression of *BUB1* was estimated by RT-qPCR. The expression levels of *BUB1* were significantly lower in si*BUB1*-transfected cells than in the siNC group in both BCa cell lines (*p* < 0.05 each); the knockdown efficiency was 90–95% (Figure 4A,B). Flow cytometry analysis of BCa cell lines indicated that the number of si*BUB1*-transfected cells in the G2/M phase was significantly increased (*p* < 0.05; Figure 4C,D), suggesting that the knockdown of *BUB1* inhibited the G2/M phase transition. These results indicate that *BUB1* caused abnormal regulation of the cell cycle during the G2/M phase, leading to the over-proliferation of cells and the accumulation of abnormal cancer cell numbers, which may underlie the NMIBC pathogenesis and progression.

## 3. Discussion

NMIBC is a tumor with an extremely heterogeneous biological behavior. Up to 80% of patients experience recurrence, and up to 50% show stage progression even after complete endoscopic resection [18]. Low-grade Ta tumors have a high recurrence rate, but rarely represent a threat to the patient. By contrast, high-grade T1 tumors show increased rates of progression, which lead to high cancer-specific death rates. Thus, the successful management of NMIBC depends on the accurate prediction of prognosis, as well as the proper selection and administration of treatment. The prediction of prognosis and patient stratification are based on clinicopathological characteristics. However, this approach is subject to inter-observer variation, which may cause misdiagnosis and thus unsuitable treatments. Molecular classification methods hold great promise for understanding the disease and designing personalized therapeutics. The aim of the present study was to identify an accurate molecular biomarker for NMIBC prognosis.

The results of the present study show that the expression of *BUB1* was higher in NMIBC than in normal controls (all *p* < 0.05). These results were verified in three independent cohorts, including RNA-seq-based data from the screening cohort, RT-qPCR-based data from the training cohort, and two public datasets (GSE13507 and E-MTAT-4321) from the validation cohort (Figure 1, Figure 2, Figure 3 and Figure S3). These analyses suggest that there is a causal relationship between the upregulation of *BUB1* and NMIBC tumorigenesis. Moreover, a high expression of *BUB1* correlated with worse clinicopathological features, including a high grade and T1 stage, and with shorter PFS of patients with NMIBC in the training and validation cohorts (all *p* < 0.05). The risk of progression was higher in NMIBC patients with higher *BUB1* expression than in those with lower *BUB1* expression, as determined by univariate Cox regression analysis in the training and validation cohorts (range of HR, 5.7–15; all *p* < 0.05; Table 1 and Table 2). In particular, the HR for *BUB1* expression was higher than that for tumor grade in the training cohort (6.076 vs. 5.508; *p* < 0.05; Table 1). A survival plot was generated to analyze the correlation between NMIBC progression and the combination index of tumor grade and *BUB1* expression. The Kaplan–Meier estimator indicated that NMIBC patients with a low grade and lower *BUB1* expression had the longest PFS, whereas patients with a high grade and higher *BUB1* expression had the shortest PFS in the training cohort, as determined after a long-term follow-up period in two validation cohorts (log-rank test, *p* < 0.05; Figure 1, Figure 2 and Figure 3). The UROMOL cohort from the E-MTAB-4321 dataset indicated that NMIBC patients with stage Ta and lower *BUB1* expression had a good prognosis, whereas patients with stage T1 and higher *BUB1* expression had a poor prognosis (log-rank test, *p* < 0.0001; Figure 3). However, analysis of the Chungbuk National University Hospital (CBNUH) microarray cohort from the GSE13507 dataset showed no differences in PFS between different combination indexes (tumor stage or grade with *BUB1* expression; data not shown). Taken together, these results support the prognostic power of *BUB1* expression for predicting NMIBC progression, which could assist clinicians in designing optimal and timely treatments.

Dysregulation of the cell cycle leads to the abnormal proliferation and division of cells, especially tumor cells [19,20]. The chromosomes replicated and were evenly distributed into daughter cells during the M phase of a cell cycle [21]. However, imprecise segregation of chromosomes can lead to the loss or gain of whole chromosomes, a status referred to as aneuploidy [21,22]. Aneuploidy is observed in over 80% of human cancers, and is associated with poor clinical outcomes [23,24]. Aberrant expression or mutation of *BUB1*, which plays a crucial role in mitosis, is an important cause of aneuploidy [10,25]. *BUB1* is a core component of the mitotic checkpoint, which is a surveillance system developed by eukaryotic cells to ensure faithful chromosome segregation, and this checkpoint could delay anaphase onset until all kinetochores are properly attached to the microtubules emanating from opposite spindle poles [5,26]. Abnormal BUB1 levels could affect the mitotic checkpoint, resulting in aberrant cell cycle progression and the conversion of normal cells into malignant cells. In the present study, *BUB1* prevented G2/M cell cycle arrest in BCa cells and promoted the proliferation of two BCa cell lines (*p* < 0.05; Figure 4). This could be attributed to *BUB1*-induced aneuploidy of BCa cells during the G2/M phase, although the exact morphological alterations of BCa cells were not analyzed. Future studies should address this issue and examine whether high *BUB1* levels can drive neoplastic transformation and promote tumor aggressiveness.

The deregulation of *BUB1* expression occurs at a higher incidence than mutations in *BUB1*. *BUB1* expression is decreased in lung, colon, and pancreatic tumors [27,28]. However, the upregulation of mitotic factors is a more common episode in human cancers. *BUB1* gene levels were found to be overexpressed in the breast and gastric cancers, as well as in lymphomas [29,30,31]. *BUB1* was identified as a prospective prognostic marker whose upregulation is associated with poor clinical outcomes in diverse tumor types [8,32,33,34]. These results support the value of the present study for explaining the role of *BUB1* as an oncogene in BCa.

In conclusion, the present findings provide additional evidence that *BUB1* acts as an oncogene in BCa, and could thus serve as an independent prognostic marker for the stratification of NMIBC patients according to favorable and poor prognoses.

## 4. Materials and Methods

### 4.1. Study Design

**Figure 5:**
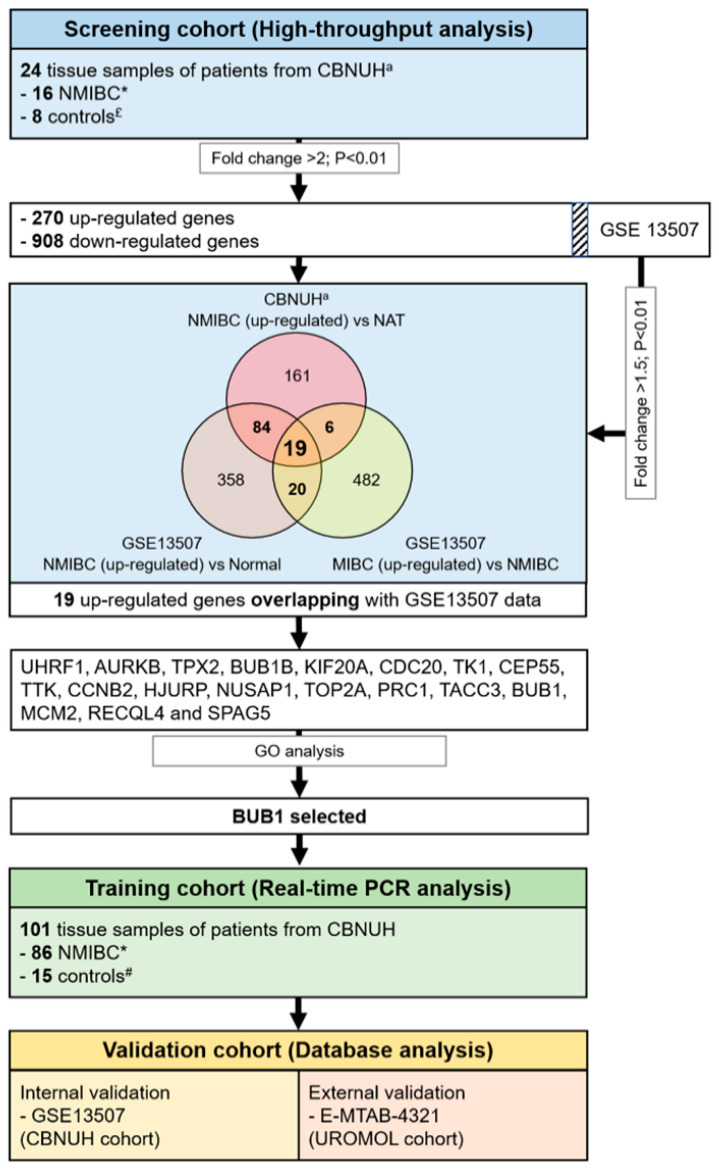
Overall study design. ^a^ RNA-seq analysis. * Cases histologically verified as urothelial carcinoma. To reduce confounding factors affecting the analyses and to delineate a more homogeneous study population, patients diagnosed with other cancers before or after urothelial carcinoma diagnosis were excluded. Tumors were staged as Ta or T1 according to the 2017 TNM Classification. ^£^ Normal adjacent tissue, NAT. ^#^ Normal bladder mucosae.

Figure 5 describes the workflow and overall study design. RNA-seq analysis was performed to screen candidate genes that were differentially expressed in patients with NMIBC (*n* = 16) and in normal controls (*n* = 8). The analysis identified 270 upregulated and 908 downregulated genes that met the conditions of fold change >2 and *p* < 0.01. A comparison with 969 genes identified in a previous profiling study of BCa [35] showed that 19 differentially expressed genes in BCa versus normal tissues overlapped with the present data (*UHRF1*, *AURKB*, *TPX2*, *BUB1B*, *KIF20A*, *CDC20*, *TK1*, *CEP55*, *TTK*, *CCNB2*, *HJURP*, *NUSAP1*, *TOP2A*, *PRC1*, *TACC3*, *BUB1*, *MCM2*, *RECQL4*, and *SPAG5*). Analysis of the biological features of the 19 genes using GO terms showed that they are involved in important steps of the cell cycle, such as nuclear division, spindle organization, and cytokinesis (Supplementary Figure S1). Among the identified genes, *BUB1*, a novel gene in BCa, was selected for further validation. In the training cohort, the mRNA expression of *BUB1* in NMIBC patients (*n* = 86) and normal controls (*n* = 15) was estimated using real-time PCR. The prognostic power of *BUB1* in NMIBC was assessed in an internal validation cohort (GSE13507) and an external validation cohort (E-MTAB-4321).

### 4.2. Statistical Analysis

Continuous variables were expressed as the mean ± standard deviation. The normality of the data was estimated by one-sample Kolmogorov–Smirnov tests. Welch’s *t*-test was used for the statistical analysis of two non-paired samples, and a paired *t*-test was used for the statistical analysis of paired samples. Error bars indicate the standard error of the mean. The groups were compared in terms of noncontinuous variables by the Chi-squared test. The values of gene expression were natural log-transformed to make the skewed data more normally distributed and to achieve constant variance. The Mann–Whitney U-test was used to examine the expression of *BUB1* in NMIBC tissues versus control tissues by real-time PCR. Univariate and multivariate Cox proportional hazard regression models were used to evaluate the prognostic significance of the clinicopathological variables. Relative risk was determined by HRs and 95% CIs. Kaplan–Meier survival curves were plotted to determine the prognostic value of the genetic biomarker and compared using the log-rank test. Statistical analyses were performed using GraphPad Prism 8 (GraphPad Software, San Diego, CA, USA) and IBM SPSS Statistics ver. 24.0 (IBM Co., Armonk, NY, USA). Specifically, the box plots and plots of the cell experiment were depicted using GraphPad Prism 8, and the survival plots were depicted using GraphPad Prism 8 and IBM SPSS Statistics ver. 24.0. *p*-values < 0.05 were considered significant.

Other materials and methods can be seen in online-only supplementary files (Supplementary materials and methods, online only).

## Figures and Tables

**Table 1:** Univariate and multivariate Cox regression analyses of factors predicting NMIBC progression.

Variables	Univariate Cox Analysis		Multivariate Cox Analysis	
	HR (95% CI)	*p*-Value	HR (95% CI)	*p*-Value
Age <65 (Ref.) vs. >65	1.946 (0.661–5.722)	0.227		
Gender Male (Ref.) vs. Female	1.872 (0.520–6.737)	0.337		
Tumor size ≤1 cm (Ref.) vs. 2–3 cm				
	1.563 (0.476–4.536)	0.670		
Multiplicity				
Single	Ref.			
2–7 >7	1.664 (0.437–6.326) 5.178 (0.890–30.116)	0.455 0.067		
2004 WHO grade Low (Ref.) vs. High				
	5.808 (2.045–16.493)	0.001 *	4.629 (1.593–13.450)	0.005 *
Stage Ta (Ref.) vs. T1				
	0.765 (0.261–2.243)	0.626		
BCG No (Ref.) vs. Yes				
	1.415 (0.474–4.225)	0.534		
*BUB1* expression Low expression (Ref.) vs. High expression	6.076 (1.367–27.007)	0.018 *	4.642 (1.021–21.097)	0.047 *

BCG, Bacillus Calmette–Guerin; CI, confidence interval; HR, hazard ratio; Ref., reference. * *p* < 0.05.

**Table 2:** Univariate Cox regression analyses to predict NMIBC progression in the validation cohorts.

*BUB1* Expression Low Expression (Ref.) vs. High Expression	Univariate Cox Analysis	
	HR (95% CI)	*p*-Value
Internal validation cohort ^a^ (*n* = 103)	15 (1.9–115)	0.011 *
External validation cohort ^b^ (*n* = 450)	5.7 (2.2–15)	<0.001 *

^a^, GSE13507 dataset; ^b^, E-MTAB-4321. CI, confidence interval; HR, hazard ratio; Ref., reference. * *p* < 0.05 was considered significant.

## Data Availability

The data presented in this study are available on request from the corresponding author.

## Supplementary Materials

The following are available online at https://www.mdpi.com/article/10.3390/ijms222312756/s1.
