# Supplementary material for: Prognostic Value of BUB1 for Predicting Non-Muscle-Invasive Bladder Cancer Progression
Source: Int J Mol Sci. 2021 Nov 25;22(23):12756. doi: 10.3390/ijms222312756 (PMC8657483; doi:10.3390/ijms222312756)
Supplement: Supplementary file 1 [file ijms-22-12756-s001.zip › ijms-1468900-supplementary.pdf]

International Journal of
Molecular Sciences

Supplementary Materials and Methods

1. Patients and tissue samples

The study methodologies conformed with the standards set by the Declaration of Helsinki. Supplementary Table S1 shows the baseline characteristics of the case subjects used for RNA-seq analysis. Of 24 bladder tissue samples collected between September 2001 and September 2014, 16 were non-muscle invasive bladder cancer (NMIBC) and eight normal adjacent tissue (NAT) samples were used as the control set. A total of 101 bladder tissue samples collected between November 1999 and June 2010 were used for RT-qPCR analysis; the baseline characteristics of the patients are listed in supplementary Table S2. Of the 101 tissue samples, 86 were obtained from primary NMIBC patients and were histologically verified as transitional cell carcinomas; the remaining 15 used as controls were normal bladder mucosae obtained from patients with suspected cancer who were ultimately diagnosed with benign diseases such as stress urinary incontinence or unknown hematuria. The biospecimens were obtained from Chungbuk National University Hospital (CBNUH; Cheongju, South Korea), which is involved in the National Biobank of Korea. The study was approved by the Institutional Review Board at CBNUH (GR2010-12-010 and GR2020-07-018), and the experiments were performed after obtaining written informed consent from all participants. To reduce the chances of confounding factors affecting the analyses, patients diagnosed with concomitant carcinoma in situ or those with carcinoma in situ lesions alone were excluded. Fresh tissues were obtained during surgical resection of transitional cell carcinoma at CBNUH. All tumors were macro-dissected, typically within 15 min of surgical resection. Each specimen was confirmed by pathological analysis of a section of fresh-frozen tissue obtained from transurethral resection of bladder tumor. Tumors were staged according to the 2017 TNM classification and graded using the 2004 WHO classification according to standard criteria [1]. Each patient was followed and managed according to standard guidelines [2-4]. Surveillance was performed by cystoscopy and upper urinary tract imaging in accordance with European Association of Urology guidelines 1-4. Recurrence was defined as relapse of primary NMIBC of the same pathologic stage, and progression of NMIBC was defined as TNM stage progression after disease recurrence. The mean follow-up period for NMIBC patients was 71.15 months (range, 10.70–174.90 months).

2. Public expression profiles

Expression profiles of the GSE13507 CBNUH microarray cohort were downloaded from the GEO online database (https://www.ncbi.nlm.nih.gov/geo/). The E-MTAB-4321 UROMOL cohort was downloaded from EMBL-EBI (http://www.ebi.ac.uk/).

3. BCa cell lines

The BCa cell lines 5637 and T24 were obtained from the Korean Cell Line Bank (KCLB, Seoul, South Korea) and all human cell lines have been authenticated using STR profiling within the last three years. Both cell lines were routinely cultured at 37°C in a humidified 5% CO2 incubator in RPMI 1640 supplemented with 10% fetal bovine serum (both from Gibco; Thermo Fisher Scientific, Waltham, MA, USA) and 1% penicillin-streptomycin (Gibco, Thermo Fisher Scientific, Waltham, MA, USA).

4. RNA extraction

Total RNA was extracted from tissues using the TRIzol reagent (Invitrogen, Carlsbad, CA, USA) as described previously [5] and stored at -80°C. The First Strand cDNA Synthesis Kit (Clontech, TAKARA, Otsu, Japan) was used for synthesizing cDNA from 1 μg total RNA according to the manufacturer's protocol.

Total RNA was extracted from cells using Ribospin II (GeneAll, Seoul, Korea), and 500 ng total RNA was reverse transcribed using the RevertAid First Strand cDNA Synthesis kit (Thermo Fisher Scientific, Waltham, MA, USA).

5. RNA-sequencing analysis

FASTQ files from sequencing experiments were processed for quality, and Trim Galore was used to remove the adapter sequences. The processed raw reads were aligned to the human genome (hg38) with STAR [6], and the aligned reads were converted into tag directories using HOMER [7]. Each file was quantified using the "analyzeRepeats" script of HOMER. Normalization of the expression levels of genes in each sample was performed by fragments per kilobase of transcript per million mapped reads (FPKM). Differentially expressed genes (DEGs) were identified using raw read counts by DESeq2 [8] analysis through the "getDifferentialExpression" command of HOMER at an adjusted P-value <0.01 and fold change >2. Metascape was used for the gene ontology (GO) analysis [9], and gene set enrichment analysis (GSEA) was performed to determine DEGs that were enriched in gene lists extracted from MSigDB [10] to determine enrichment in gene sets from curated (C2) collections.

6. RT-qPCR

Tissue mRNAs were amplified by real-time RT-qPCR using a Rotor-Gene 6000 instrument (Qiagen, Hilden, Germany), and the relative gene expression was quantified using the 2-ΔΔCt method [11]. SYBR Premix Ex Taq Ⅱ (Clontech, TAKARA, Otsu, Japan) was used for real-time PCR according to the manufacturer's protocol. The PCR reaction was performed in a final volume of 10 μL containing 5 μL of 2× SYBR Premix Ex Taq buffer, 0.5 μL of each 5′ and 3′ primer (10 pM/μL), and 2 μL sample cDNA. A known concentration of the PCR product was 10-fold serially diluted from 100 pg/μL to 0.1 pg/μL and used to establish a standard curve. The real-time PCR conditions were as follows: 1 cycle at 96°C for 20 sec, followed by 40 cycles of 3 sec at 96°C for denaturation; 15 sec at 63°C for annealing; and 15 sec at 72°C for extension. The melting program was performed at 72–95°C at a heating rate of 1°C per 45 sec. Rotor-Gene Q software 2.3.1.49 was used for capturing and analyzing spectral data. All samples were run in triplicate. Gene expression was normalized to the expression of GAPDH. The following primers were used to amplify candidate genes: BUB1 (Gene ID: 699), sense, 5′-AGCCCAGACAGTAACAGACTC-3′ (21 bp, Tm 64℃); antisense, 5′-GTTGGCAACCTT ATGTGTTTCAC-3′ (23 bp, Tm 66℃). The amplicon size was 113 bp. The control GAPDH (Gene ID: 2597) primers were as follows: sense, 5′-CATGTTCGTCATGGGTGTGA-3′ (20 bp, Tm 60 ℃); antisense, 5′-ATGGCATGGACTGTGGTCAT-3′ (20 bp, Tm 60℃). The amplicon size was 156 bp.

Gene expression in cells was evaluated in replicates with TOPreal qPCR 2× PreMIX (SYBR Green with low ROX; Enzynomics, Daejeon, Korea). Rotor-Gene Q software 2.3.1 was used for capturing and analyzing spectral data. Gene expression was normalized to the expression of TBP. The following primers were used to amplify candidate genes: BUB1 (Gene ID: 699), sense, 5′-AGCCCAGACAGTAACAGACTC-3′ (21 bp, Tm 60.9℃); antisense, 5′-GTTGGCAACCTTATGTGTTTCAC-3′ (23 bp, Tm 60.5℃). The amplicon size was 136 bp. The control TBP (Gene ID: 6908) primers were as follows: sense, 5′-CCCGAAACGCCGAATATAATCC-3′ (22 bp, Tm 61.2 ℃); antisense, 5′-AATCAGTGCCGTGGTTCGTG-3′ (20 bp, Tm 63℃). The amplicon size was 80 bp.

7. SiRNA-mediated knockdown

Human BUB1 gene knockdown was achieved using the SMART pool ON-TARGETplus siRNA (Dharmacon: L-004102-00-0005). The ON-TARGETplus non-targeting pool was used as a negative control (Dharmacon: D-001810-10-05). The 5637 and T24 cell lines were seeded into 6-well plates, incubated for 24 h, and then transfected with RNA duplex and Lipofectamine RNAi-MAX (Invitrogen; Thermo Fisher Scientific, Waltham, MA, USA) in Opti-MEM I reduced serum medium (Thermo Fisher Scientific, Waltham, MA,

USA) without antibiotics and following the manufacturer's instructions. The medium was changed into complete medium with antibiotics after 24 h, and the cells were harvested for further experiments after 24 h.

8. Flow cytometry

The 5637 and T24 cell lines were transfected with non-targeting control siRNA (siNC) and BUB1 siRNA (siBUB1). After 48 h, the cells were harvested. For detection of the cell cycle, cells were fixed in ice-cold 70% ethanol with PBS and stained with Propidium Iodide (PI) solution (Invitrogen; Thermo Fisher Scientific, Waltham, MA, USA). Cells were analyzed for DNA content by flow cytometry using a CytoFLEX (Beckman Coulter, Brea, CA, USA). The data were plotted using FlowJo software (TreeStar).

References

1. Babjuk M, Böhle A, Burger M, Compérat E, Kaasinen E, and Palou J. EAU Guidelines on Non-muscle invasive (Ta, T1, CIS) Bladder Cancer. EAU guidelines 2020.
2. Oosterlinck W, Lobel B, Jakse G, et al. Guidelines on bladder cancer. Eur Urol **2002**, *41*,105-112.
3. Babjuk M, Oosterlinck W, Sylvester R, Kaasinen E, Böhle A, and Palou-Redorta J. EAU guidelines on non-muscle-invasive urothelial carcinoma of the bladder. Eur Urol **2008**, *54*, 303-314.
4. Babjuk M, Oosterlinck W, Sylvester R, Kaasinen E, Boehle A, and Palou-Redorta J. EAU guidelines on non-muscle-carcinoma of the bladder. Actas Urol Esp **2009**, *33*, 361-371.
5. Kim W, Kim J, Yan C, et al. S100A9 and EGFR gene signatures predict disease progression in muscle invasive bladder cancer patients after chemotherapy. Ann Oncol **2014**, *25*, 974-979.
6. Magoč T and Salzberg SL. FLASH: fast length adjustment of short reads to improve genome assemblies. Bioinformatics **2011**, *27*, 2957-2963.
7. Heinz S, Benner C, Spann N, et al. Simple combinations of lineage-determining transcription factors prime cis-regulatory elements required for macrophage and B cell identities. Mol Cell **2010**, *38*, 576-589.
8. Love MI, Huber W, and Anders S. Moderated estimation of fold change and dispersion for RNA-seq data with DESeq2. Genome Biol **2014**, *15*, 1-21.
9. Tripathi S, Pohl MO, Zhou Y, et al. Meta-and orthogonal integration of influenza "OMICs" data defines a role for UBR4 in virus budding. Cell Host Microbe **2015**, *18*, 723-735.
10. Subramanian A, Tamayo P, Mootha VK, et al. Gene set enrichment analysis: a knowledge-based approach for interpreting genome-wide expression profiles. P Natl Acad Sci **2005**, *102*, 15545-15550.
11. Livak KJ and Schmittgen TD. Analysis of relative gene expression data using real-time quantitative PCR and the 2− ΔΔCT method. Methods **2001**, *25*, 402-408.

Supplementary Figures

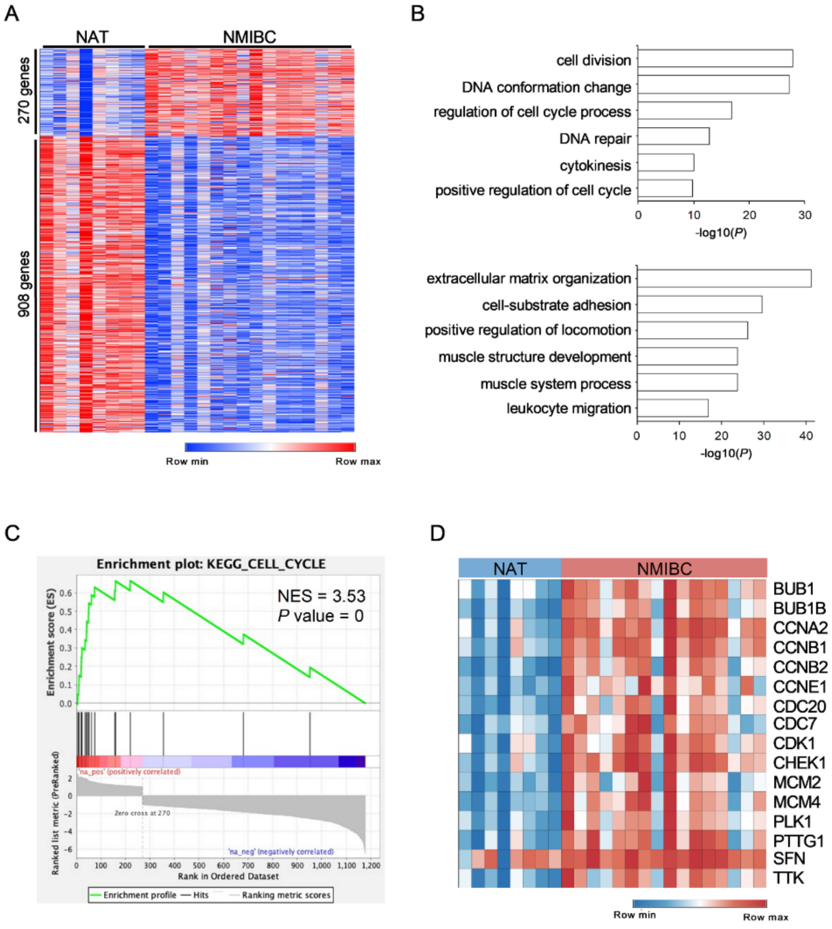

Supplementary Figure S1. RNA-seq analysis of gene expression in NMIBC. A. Heap-map clustering of 1178 DEGs between NMIBC and NAT. B. GO analysis of DEGs between NMIBC and NAT (red, upregulated genes; blue, downregulated genes). C. GSEA showed that DEGs enriched in NMIBC compared with NAT were related to cell cycle (NES, 3.53; $P < 0.0001$) processes. D. The heat-map corresponding to C shows the core genes enriched in the cell cycle pathway. DEG, differentially expressed genes; GO, Gene Ontology; GSEA, gene set enrichment analysis; NAT, normal adjacent tissue; NES, normalized enrichment score; NMIBC, non-muscle invasive bladder cancer.

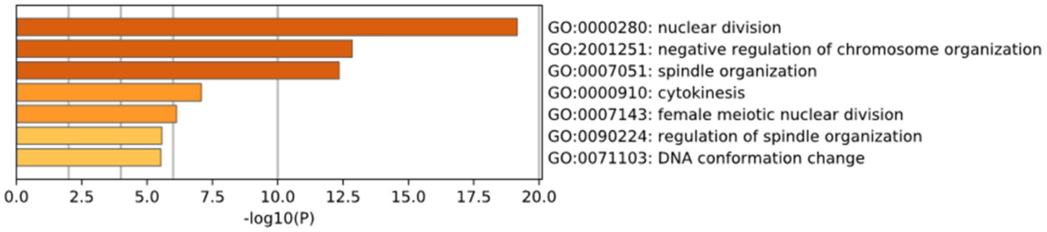

Supplementary Figure S2. GO analysis of 19 genes overlapped in the screening test. Nineteen differentially expressed genes (UHRF1, AURKB, TPX2, BUB1B, KIF20A, CDC20, TK1, CEP55, TTK, CCNB2, HJURP, NUSAP1, TOP2A, PRC1, TACC3, BUB1, MCM2, RECQL4, and SPAG5) in BCa versus normal tissues were analyzed using GO terms. The genes were involved in important steps of the cell cycle. BCa, bladder cancer; GO, Gene Ontology.

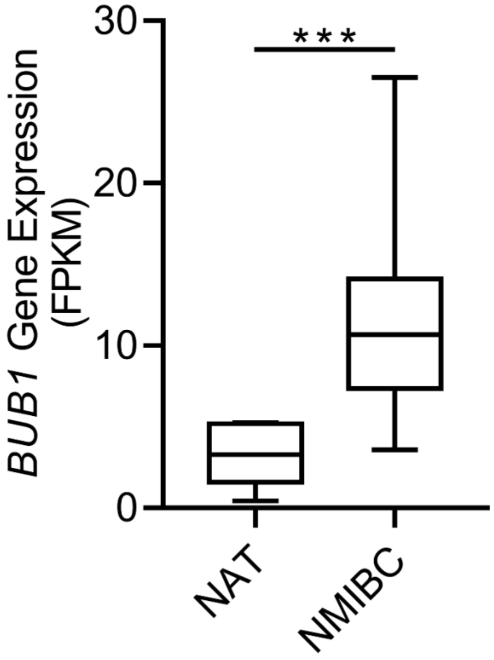

Supplementary Figure S3. Gene expression of *BUB1* in the screening cohort. *BUB1* expression was higher in patients with NMIBC than in NATs from the CBNUH RNA-seq cohort. CBNUH, Chungbuk National University Hospital; FPKM, Fragments Per Kilobase of transcripts per Million mapped reads; NAT, normal adjacent tissue; NMIBC, non-muscle invasive bladder cancer. The result is depicted as the mean with 95% CI. *P*-values were determined by Welch's t-test. ***$P < 0.001$.

Supplementary Tables

Supplementary Table S1. Clinicopathological features of NMIBC patients and controls

Characteristics	NMIBC	Control	*P*-value
**No.**	16	8	
**Mean age ± SD**	60.5 ± 10.3	60.0 ± 11.5	0.809*
**Gender (%)**			0.722#
Male	13 (81.25%)	6 (75%)	
Female	3 (18.75%)	2 (25%)	
**2004 WHO grade**			
High-grade	5 (31.25%)		
Low-grade	11 (68.75%)		
**Stage**			
Ta	11 (68.75%)		
T1	5 (31.25%)		

NMIBC, non-muscle invasive bladder cancer; SD, standard deviation. Controls comprised normal adjacent tissues.
**P*-value obtained using the Student's t-test.
#*P*-value obtained using the Chi-squared test.

Supplementary Table S2. Clinicopathological features of primary bladder cancer patient and control tissues (normal bladder mucosae) examined in this study

Variable	NMIBC	Control	*P*-value
**No.**	*86*	*15*	
**Mean age ± SD**	64.45 ± 13.86	65.53 ± 13.97	0.782*
**Gender (%)**			0.096#
Male	73 (84.9%)	16 (100%)	
Female	13 (15.1%)	0	
**Tumor size (%)**			
≤1 cm	52 (60.5%)		
2–3 cm	34 (39.5%)		
**Multiplicity (%)**			
Single	47 (54.7%)		
2–7	27 (31.4%)		
>7	12 (14.0%)		
**Grade, 2004 WHO grading system (%)**			
Low	64 (74.4%)		
High	22 (25.6%)		
**Stage (%)**			
TaN0M0	25 (29.1%)		
T1N0M0	61 (70.9%)		
**BCG therapy (%)**			
No	54 (62.8%)		
Yes	32 (37.2%)		
**Recurrence – no. of patients (%)**			
No	51 (59.3%)		
Yes	35 (40.7%)		
**Progression – no. of patients (%)**			
No	71 (82.6%)		
Yes	15 (17.4%)		
**Survival – no. of patients (%)**			

Alive	56 (65.1%)
Death	30 (34.9%)
**Mean follow-up (range) – months**	71.15 (10.70–174.90)

BCG, Bacillus Calmette-Guerin; NMIBC, non-muscle invasive bladder cancer; SD, standard deviation.
*P-value obtained using the Student's t-test.
[#]P-value obtained using the Chi-squared test.
